# Clinical features and markers to identify pulmonary lesions caused by infection or vasculitis in AAV patients

**DOI:** 10.1186/s12890-023-02317-7

**Published:** 2023-01-18

**Authors:** Yujuan Wang, Zhuan Qu, Wei Liang, Xinghua Chen, Cheng Chen, Hui Cheng, Haiyun Hu, Zhongpin Wei, Ke Su, Lianhua Yang, Huiming Wang

**Affiliations:** Department of Nephrology, Renmin Hospital of Wuhan University, Wuhan, 430060 Hubei Province People’s Republic of China

**Keywords:** ANCA-associated vasculitis (AAV), Lung lesion, Infection, Differential diagnosis

## Abstract

**Objectives:**

Pulmonary lesion is frequently seen in ANCA-associated vasculitis (AAV) patients primarily due to AAV lung involvement or infection, which are hard to differentiate due to their high similarity in clinical manifestations. We aimed to analyze the clinical features of pulmonary lesions consequent to AAV involvement or infection in AAV patients and further identify the markers for differential diagnosis.

**Methods:**

140 AAV patients who admitted to the Renmin Hospital of Wuhan University from January 2016 to July 2021 were included in this study. According to the nature of lung conditions, these patients were divided into the non-pulmonary lesion group, the lung infection group and the non-pulmonary infection group, and their demographics, clinical symptoms, imaging features, as well as laboratory findings were compared. A receiver operating characteristic (ROC) curve was drawn, and the diagnostic efficacy of single biomarker and composite biomarkers on pulmonary infection was then evaluated.

**Results:**

The patients in the lung infection group were significantly older than those in the no lesion group (63.19 ± 14.55 vs 54.82 ± 15.08, *p* = 0.022). Patients in the lung infection group presented more frequent symptoms and more obvious pulmonary image findings. Compared with patients in the non-pulmonary infection group, patients in the lung infection group showed a higher symptom incidence of fever, chest tightness, cough and expectoration, and hemoptysis (52.94% vs 16.00%, 61.76% vs 40.00%, 72.06% vs 46.00%, 27.94% vs 8.00%, *p* < 0.05, respectively), and more changes in pulmonary CT scanning images in terms of patched/striped compact opacity, alveolar hemorrhage, bronchiectasis, pleural effusion, as well as mediastinal lymphadenopathy (89.71% vs 52.00%, 11.76% vs 2.00%, 22.06% vs 8.00%, 50.00% vs 20.00%, 48.53% vs 24.00%, *p* < 0.05, respectively). In addition, patients in the lung infection group had significantly higher levels of serum pro-calcitonin (PCT), C-reactive protein (CRP), amyloid A (SAA), blood neutrophil-to-lymphocyte ratio (NLCR), erythrocyte sedimentation rate (ESR), as well as Birmingham vasculitis activity score (BVAS) than patients in the other two groups (*p* < 0.05). Among all biomarkers, PCT exhibited the highest diagnostic efficacy (0.928; 95%CI 0.89–0.97) for pulmonary infected AAV patients at a cut-off score of 0.235 ng/ml with 85.3% sensitivity and 84% specificity. Moreover, the composite biomarker of PCT-CRP-NLCR showed more diagnostic efficacy (0.979; 95% CI 0.95–1.00) in distinguishing the infectious and non-infectious lung injuries in AAV patients.

**Conclusions:**

AAV patients with lung infection manifested more clinical symptoms and prominent lung image changes. The PCT and composite biomarker PCT-CRP-NLCR showed high diagnostic efficacy for a lung infection in AAV patients. Pulmonary lesion caused by either infection or AAV involvement is commonly seen and difficult to distinguish. We aim to identify the biomarkers that can be applied in the differentiation diagnosis of pulmonary lesions in AAV patients.

**Supplementary Information:**

The online version contains supplementary material available at 10.1186/s12890-023-02317-7.

## Background

Anti-neutrophil cytoplasmic antibodies (ANCA)-associated vasculitis (AAV) is a kind of systemic disease characterized by extensive lesions of inflammation and fibrin necrosis in small blood vessel walls. Three clinical types of AAV, including microscopic polyangiitis (MPA), granulomatosis with polyangiitis (GPA), and eosinophilic granulomatosis with polyangiitis (EGPA) have been reported [1]. AAV usually implicated multiple organs and tissues, among which, kidneys and lungs are the most frequently affected, with an incidence of 80–100% [2] and 62–85% [3, 4] respectively. ANCA-associated glomerulonephritis (AAGN) is the primary cause of acute kidney injury in elderly patients and is conducive to serious conditions and a low survival rate [5]. Pulmonary disease is another major complication that seriously threatens AAV patients and imposes thorny challenges to doctors' diagnosis and treatment. The reason is that, clinically, pulmonary vasculitis and pulmonary infection in AAV patients are often intertwined and difficult to distinguish. The clinical and imaging manifestations of AAV lung involvement are diverse and atypical. At the same time, AAV patients are often complicated with lung infection due to low immune function, which increases the complexity of lung injury and the difficulty of differential diagnosis.

Whether it is a lung infection or lung injury caused by vasculitis, patients can show symptoms of fever and lung shadows in radiological images [6]. It has been reported that the misdiagnosis rate of lung infection in AAV patients ranged from 17 to 26% [7]. Accurately identifying the nature of lung injury in patients with AAV is very important because unreasonable treatment based on the wrong diagnosis may lead to serious consequences. For instance, missed diagnosis of a lung infection may prompt doctors to use immunosuppressants without giving the necessary antibiotics, which may eventually lead to catastrophic infection out of control. In contrast, misdiagnosing pulmonary vasculitis injury as infection may delay the prescription of immunosuppressive agents, which will lead to the progression of AAV-associated kidney or other tissue injuries. To avoid the improper treatment of pulmonary complications in AAV patients, it is urgent to identify reliable markers to distinguish the pulmonary lesions of infection and vasculitis. Recently, a panel of biomarkers, including neutrophil-to-lymphocyte ratio (NLCR), serum amyloid A (SAA), as well as pro-calcitonin (PCT) have been used to identify and assess the infectious status in clinics [8, 9]. However, to our knowledge, similar markers for assessing the infection of AAV patients have not yet been fully explored.

In this study, we retrospectively studied the clinical features of 140 AAV patients who had been admitted to our hospital from January 2016 to July 2021. All patients presented with pulmonary diseases caused either by infection or AAV involvement. The clinical manifestations, pulmonary radiographic features, and laboratory findings were reviewed, and comparative analyses on patients with lung infection or not were performed to explore the reliable markers for differential diagnosis of pulmonary infection and vasculitis injury in AAV patients.

## Methods

### Patients and study design

268 patients that were admitted and initially diagnosed with AAV in the Renmin Hospital of Wuhan University from January 1, 2016, to July 1, 2021, were enrolled. AAV diagnosis was made following the criteria of the 2012 Chapel Hill Consensus Conference [1] and also referred to the 2022 ACR/EULAR classification criteria [10–12]. AAV activity was assessed based on the Birmingham vasculitis activity score (BVAS) system, 2008 version [13].

Patients were excluded if any of the following conditions exist: Received treatment of antibiotics, hormones, or immunosuppressive agents within 3 months before the enrollment; Superimposed with other connective tissue diseases; with other concurrent conditions such as systemic infections, pulmonary overhydration, tuberculosis, or lung tumors; Data in the medical record are incomplete.

The enrolled patients were classified into two major groups according to whether pulmonary lesions were found by lung CT manifestation: the non-pulmonary lesion (NL) group and the pulmonary lesion (PL) group. Patients in the PL group were further divided into the lung infection group (LI) and the non-pulmonary infection group (NI) group based on the presence or absence of lung infection. Lung infection could be diagnosed if (1) pathogenic microorganisms were detected in patients' sputum, alveolar lavage fluid, pleural effusion, and other body fluids through pathogenic microorganism culture or other pathogenic detection methods [14], and the category of pathogens was shown in Additional file 1: Table S2; or (2) although there was no etiological evidence, the symptoms of the patients were significantly relieved and the scope of chest CT lesions was significantly reduced after receiving standard antimicrobial treatment. A comprehensive analysis of the patient's course of disease supported the existence of pulmonary infection. Non-lung infection was determined if (1) the patient’s pulmonary symptoms were rapidly relieved and the scope of the lesion was significantly reduced after receiving therapy of glucocorticoids steroids or other immune-suppressive agents, which was an adjustment by replacing the ineffective treatment of standard anti-biotics in the previous stage, and use of antibiotics at that time was a diagnostic treatment for the possible lung infection although no pathogen evidence was found in the patients; or (2) patients were primarily diagnosed with AAV lung involvement and showed improvement after direct treatment with hormones and/or immunosuppressive agents without the use of anti-infective drugs.

### Clinical and laboratory data

Patients’ general information (age, gender), clinical manifestations at admission (including fever, cough, sputum, hemoptysis, fatigue, edema), organ involvement (including kidneys, digestive system, heart, skin/mucosa, eyes, ears, nose, throat, and nervous system), pulmonary imaging appearances, and laboratory findings were collected. The main laboratory parameters included blood neutrophil-to-lymphocyte ratio (NLCR), serum pro-calcitonin (PCT), C-reactive protein (CRP), amyloid A (SAA), erythrocyte sedimentation rate (ESR), albumin (ALB), platelet count (PLT), estimated glomerular filtration rate (eGFR), cANCA, pANCA, myeloperoxidase (MPO), and proteinase-3 (PR3). The patients were scored using BVAS based on their conditions and organ involvement, and the details regarding the study populations are listed in Additional file 1: Table S1.

### Statistical analysis

All data were statistically analyzed using SPSS 20.0 software. All measurement data that conformed to the normal distribution were expressed as mean ± standard deviation (x ± SD) and compared using a t-test. All measurement data that did not conform to the normal distribution were expressed as median (P25, P75) and compared using the Mann–Whitney U test. All count data were expressed as the number of cases plus a percentage (%) and compared using the Chi-square test. The diagnostic performance of PCT, SAA, and other laboratory parameters for lung infection was evaluated using the receiver operating characteristic curve (ROC). A composite indicator was constructed using the logistic regression model with whether AAV was complicated with lung infection as the binary variable. By comparing the area under the curve (AUC) of the composite indicator and the original indicators, the best cut-off value was determined at the largest Youden index, and the sensitivity and specificity were calculated. The ROC curves were compared using MedCalc. The factors related to lung infection in AAV patients were analyzed using the binary logistic regression analysis module. *p* < 0.05 was considered statistically significant.

### Ethical approval

The study was conducted complying with the basic principles of medical ethics and was approved by the Ethics Committee of Renmin Hospital of Wuhan University (Approval Code: WDRY2020-k064; Approval Date: 2020.02.25). Written informed consent for inclusion from each patient was waived by the Ethics Committee of Renmin Hospital of Wuhan University because this was a retrospective study, and no study-related interventions were included.

## Results

### General characteristics

**Fig. 1:**
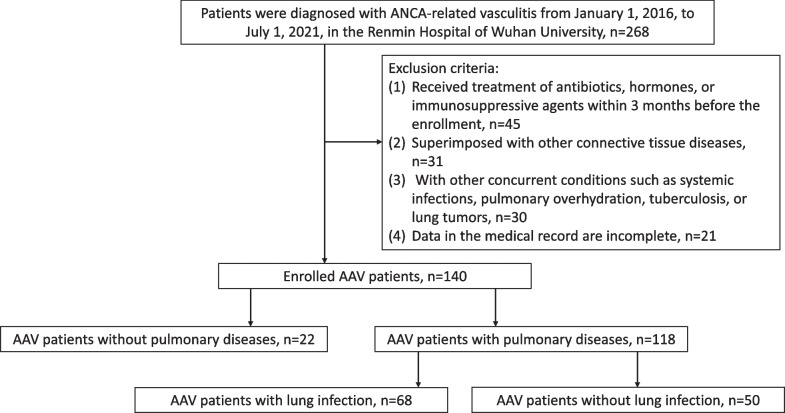
Study flowchart of patient’s enrolling and data analysis. 140 AAV patients met the inclusion and exclusion criteria were enrolled in this study. The patients were stratified according to the nature of the pulmonary disease. Demographic and clinical features were collected and analyzed

A total of 140 newly diagnosed AAV patients who met the inclusion criteria were included. Among them, 22 had no pulmonary lesions and were assigned to the NL group, and 118 had pulmonary lesions and were assigned to the PL group. Of the 118 AAV patients in the PL group, 68 (57.63%) were complicated with a lung infection and were assigned to the LI group, and 50 (42.37%) had no lung infection and were assigned to the NI group (Fig. 1).

The clinical characteristics of patients in the three groups were summarized in Table 1. As it showed that patients in the LI group were significantly older than patients in the NL group (63.19 ± 14.55 vs. 54.82 ± 15.08, *p* = 0.022). In addition, patients in the LI group had significantly higher PCT, CRP, SAA, NLCR, ESR, and BVAS and significantly lower ALB than patients in the NL and NI groups (*p* < 0.05). Among the 140 patients, 120 (85.71%) had kidney involvement, 118 (84.29%) had lung involvement, 6 (4.29%) had skin and mucous membrane involvement, 14 (10.00%) had ear, nose, and throat involvement, 20 (14.29%) had digestive system involvement, 7 (5.00%) had heart involvement, and 1 (0.71%) had nervous system involvement. The proportion of patients complicated with cardiac involvement in the LI group was significantly higher than that in the NL and NI groups (*p* < 0.05). The proportion of patients with involvement of other organs showed no significantly different among the three groups (*p* > 0.05).

**Table 1:** Demographic and clinical characteristics of the subjects

	NL group n = 22	NI group n = 50	LI group n = 68
Demographic data			
Age (years)	54.82 ± 15.08	59.08 ± 12.99	63.19 ± 14.55*
Sex (M/F)	12/10	30/20	34/34
Laboratory data			
PCT (ng/ml)	0.12 ± 0.27	0.15 ± 0.20	3.73 ± 6.42**
CRP (mg/l)	5.45 ± 13.83	13.26 ± 21.80	113.74 ± 63.05**
SAA(mg/L)	27.14 ± 49.87	43.12 ± 59.35	193.57 ± 71.71**
NLCR	4.13 ± 3.03	3.63 ± 2.09	15.59 ± 26.12***
Neutrophil counts (× 10^9^/L)	4.63 ± 1.45	4.65 ± 2.41	9.99 ± 3.68**
Lymphocyte counts (× 10^9^/L)	1.43 ± 0.61	1.41 ± 0.54	1.07 ± 0.54
ESR(mm/h)	49.09 ± 26.95	42.00 ± 33.27	71.36 ± 33.20**
HGB (g/L)	93.18 ± 26.20	95.14 ± 25.65	89.24 ± 20.31
PLT (× 10^9^/L)	215.18 ± 87.42	233.12 ± 96.48	269.44 ± 112.17*
ALB (g/L)	34.66 ± 5.11	34.05 ± 4.78	31.69 ± 5.11**
eGFR (ml/min/1.73 m^2^)	30.66 ± 26.65	43.66 ± 44.67	36.95 ± 39.63
pANCA positive rate (N(%))	19 (86.36%)	41 (82.00%)	55 (80.88%)
cANCA positive rate (N(%))	3 (13.64%)	9 (18.00%)	13 (19.12%)
MPO-ANCA (RU/mL)	33.65 ± 33.76	58.08 ± 43.14*	49.25 ± 43.54
PR3-ANCA (RU/mL)	2.64 ± 2.94	6.64 ± 17.16	12.83 ± 30.14*
Subtype of the AAV patients (N(%))			
GPA	3 (13.64%)	9 (18.00%)	13 (19.12%)
MPA	19 (86.36%)	41 (82.00%)	54 (79.41%)
EGPA	0 (0%)	0 (0%)	1 (1.47%)
Organ involvement (N(%))			
Cutaneous	0 (0.00%)	3 (6.00%)	3 (4.41%)
Mucous membrane and eye	1 (4.55%)	1 (2.00%)	2 (2.94%)
Ear, nose, and throat	1 (4.55%)	2 (4.00%)	11 (16.18%)
Renal	20 (90.91%)	43 (86.00%)	57 (83.82%)
Cardiovascular	0 (0.00%)	0 (0.00%)	7 (10.29%)**
Abdominal	2 (9.10%)	6 (12.00%)	12 (17.65%)
Nervous system	0 (0.00%)	0 (0.00%)	1 (1.47%)
BVAS	12.01 ± 1.23	13.56 ± 4.66*	19.06 ± 5.59**

Values are expressed as mean ± standard deviation or number (percentage)

eGFR, estimated glomerular filtration rate; BVAS, Birmingham Vasculitis Activity Score; GPA, granulomatosis with polyangiitis; MPA, microscopic polyangiitis; EGPA, eosinophilic granulomatosis with polyangiitis; MPO, myeloperoxidase; PR3, proteinase 3; PCT, pro-calcitonin; CRP, C-reactive protein; SAA, serum amyloid A; NLCR, neutrophil-to-lymphocyte ratio; ESR, erythrocyte sedimentation rate

^*^p < 0.05 vs NL group, **p < 0.05 vs both NI group and NL group

The positive rate of ANCA displayed no significant deference among the three groups, but patients in the LI group had significantly higher PR3 titer than patients in the NL group, and patients in the NI group had significantly higher MPO titer than patients in the NL group (*p* < 0.05).

### Pulmonary-related clinical and radiographic image manifestations

Among the 118 patients in the PL group, 68 (57.63%) had lung infections. The most common clinical manifestation of the 118 patients was fatigue (84/118, 71.19%), but its incidence was not statistically different between the LI group and NI group (*p* > 0.05). The symptom incidence of fever, chest tightness, cough, and expectoration, and hemoptysis were higher in the LI group than in the NI group (52.94% vs 16.00%, 61.76% vs 40.00%, 72.06% vs 46.00%, 27.94% vs 8.00%, *p* < 0.05, respectively). Forty-one patients (34.75%) had edema, but its incidence was not statistically different between the LI and NI groups (*p* > 0.05). The duration of symptoms for patients in the NI and LI group were shown in Additional file 1: Table S3. The duration of fever was significantly longer in the LI Group than in the NI group (*p* < 0.05).

Chest CT examination showed that image appearances of patched/striped compact opacity, alveolar hemorrhage, bronchiectasis, pleural effusion, as well as mediastinal lymphadenopathy were more frequent in patients of the LI group than NI group (89.71% vs 52.00%, 11.76% vs 2.00%, 22.06% vs 8.00%, 50.00% vs 20.00%, 48.53% vs 24.00%, *p* < 0.05, respectively). The incidence of other pulmonary imaging findings was not significantly different between the LI and NI groups (*p* > 0.05) (Table 2).

**Table 2:** Clinical and imaging manifestations of the respiratory system

	NI n = 50	LI n = 68	*p* value
Clinical manifestations (N(%))			
Weakness	33 (66.00%)	51 (75.00%)	0.179
Chest tightness	20 (40.00%)	42 (61.76%)	0.019*
Cough and expectoration	23 (46.00%)	49 (72.06%)	0.004*
Hemoptysis	4 (8.00%)	19 (27.94%)	0.007*
Fever	8 (16.00%)	36 (52.94%)	0.000*
Edema	15 (30.00%)	26 (38.24%)	0.353
Imaging manifestations (N(%))			
Interstitial lesions	15 (30.00%)	14 (20.59%)	0.241
Reticular lesion	15 (30.00%)	14 (20.59%)	0.241
Honeycomb lesion	4 (8.00%)	2 (2.94%)	0.216
Alveolar hemorrhage	1 (2.00%)	8 (11.76%)	0.048*
Ground glass opacity	11 (22.00%)	11 (16.18%)	0.422
Patchy/banded high-density lesions	26 (52.00%)	61 (89.71%)	0.000*
Emphysema/bullae	16 (32.00%)	23 (33.82%)	0.835
Bronchiectasia	4 (8.00%)	15 (22.06%)	0.040*
Pulmonary nodule	22 (44.00%)	31 (45.59%)	0.864
Pleural thickening	24 (48.00%)	34 (50.00%)	0.830
Pleural effusion	10 (20.00%)	34 (50.00%)	0.001*
Mediastinal lymphadenopathy	12 (24.00%)	33 (48.53%)	0.007*

Values are expressed as number (percentage)

*p < 0.05 NI group versus LI group

### Diagnostic performance of indicated biomarkers for a lung infection in AAV patients

**Fig. 2:**
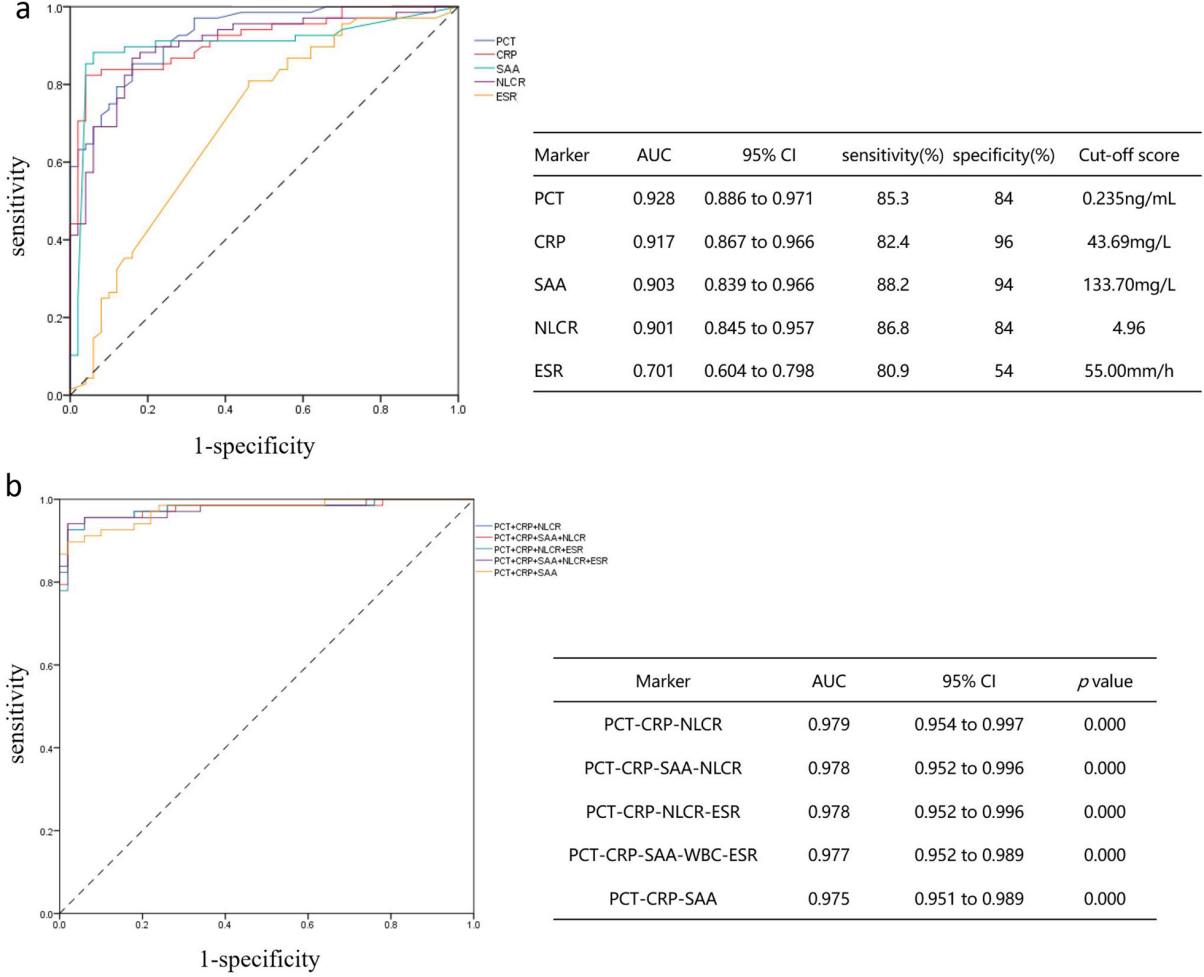
Receiver operator characteristic (ROC) curve for lung infection discrimination with AI group, and area under the ROC (AUROC) for the single (**a**) and combined biomarkers (**b**) evaluated in this study

The levels of PCT, CRP, SAA, NLCR, and ESR were significantly higher in the LI group than in the NI group (3.73 ± 6.42 vs 0.15 ± 0.20, 113.74 ± 63.05 vs 13.26 ± 21.80, 193.57 ± 71.71 vs 43.12 ± 59.35, 15.59 ± 26.12 vs 3.63 ± 2.09, 71.36 ± 33.20 vs 42.00 ± 33.27, *p* < 0.05, respectively). The ROC curves of the above indicators were drawn to evaluate their diagnostic performance. Figure 2 shows the AUC, sensitivity, specificity, and cut-off score of each indicator. Among these parameters, PCT exhibited the highest diagnostic efficacy (0.93; 95%CI 0.89–0.97) for pulmonary infected AAV patients at a cut-off score of 0.235 ng/ml with 85.3% sensitivity and 84% specificity. The AUC of all possible biomarker combinations as calculated. Figure 2b shows the AUC values of the top five biomarker combinations. The composite biomarker PCT-CRP-NLCR showed higher diagnostic efficacy (0.979; 95% CI 0.95–1.00) in distinguishing the infectious and non-infectious lung injuries in AAV patients.

## Discussion

AAV, as a systemic vasculitis, could involve multiple organs and cause damage and dysfunction, and the kidney and lung are the most affected [3, 4]. In clinical practice, nephrologists might receive patients with AAV-associated renal injury spiked with lung diseases, and feel difficult to treat these patients' lung diseases. In theory, lung infection and AAV-related pulmonary lesions may coexist and are difficult to distinguish. It is tricky when the prescription of hormones or immunosuppressants is needed to control the progress of renal injury while the patient's lung infection cannot be completely ruled out. In this study, we found that 118 (84.29%) of 140 AAV patients had pulmonary lesions, which is higher than that reported in the literature [15]. Among the 118 patients with pulmonary lesions, 68 (57.63%) patients had a lung infection, which is higher than in other studies [16, 17]. We also noted that among the enrolled subjects, 3 patients died due to uncontrolled lung infection. Our findings together with previous reports underscored the emphasis of differentiating lung infection from vasculitis lung injury in AAV patients. AAV could occur in all ages, with a high incidence in patients at the age of 50–60 years [18]. The average age of the patients with a lung infection in this study was 63 years, significantly higher than that of patients without pulmonary lesions, consistent with previous studies [15, 17], suggesting that elderly AAV patients are more prone to lung infection.

Among the 118 patients with pulmonary lesions in this study, the most common clinical manifestation was fatigue (71.19%). However, the symptom lacks specificity. The incidence of fever, chest tightness, coughing, sputum, and hemoptysis was significantly higher in the LI group than in the NI group, suggesting that complications with infection could lead to more severe clinical symptoms. In China, most AAV patients are MPA positive and show image features of lung parenchyma and interstitium. The parenchymal manifestations are mainly diffuse ground-glass opacity in both lung lobes and diffuse alveolar exudative damage, while the interstitial manifestations are mainly fiber stripe, grid and honeycomb-like shadows accompanied possibly by pleural thickening, nodules, pleural effusion, bronchiectasis and other non-specific manifestations [19, 20]. In this study, we found the incidence of non-specific manifestations such as alveolar hemorrhage, pleural effusion, patch/stripe density increase, bronchiectasis and mediastinal lymphadenopathy were significantly higher in patients of the LI group than of the NI group. However clinical and radiographic manifestations are not fully specific to the nature of lung injury and are insufficient to distinguish between lung infection and vasculitis injury.

ANCA is not only a diagnostic indicator but also a player in the pathogenesis of AAV. However, using the ANCA level to assess AAV activity or disease recurrence is still controversial [21, 22]. In this study, 82.14% of patients were pANCA positive, and ANCA positive rate was not significantly different among the three groups. PR3 titer was higher in the LI than in the NL group. MPO titer was significantly higher in the NI group than in the NL group. However, PR3 and MPO titers were not significantly different between the LI and NI groups, suggesting that patients with high PR3 and MPO may be more likely to have pulmonary lesions, but these two indexes cannot predict whether they were complicated by a lung infection.

Some biomarkers such as PCT, CRP, SAA, and ESR are helpful in the identification of infection [8, 9, 23–27], yet remain unclear for AAV patients. According to previous studies, it seems that higher CRP is more likely to indicate lung infection [24], while ESR is more likely to indicate AAV activity [28]. However, their significance in AAV patients still needs to be further explored. In recent years, NLCR has been regarded as a new inflammatory indicator. It composes of two subtypes of white blood cells, which not only indicates the role of neutrophils in infection but also is related to lymphocytes. When the body is infected, this indicator is more effective than white blood cell count and can more accurately and truly reflect the degree of infection [29, 30]. In this study, we compared the serum levels of the above indicators in each group and found that the above indicators were significantly higher in the LI but showed no significant difference between the NI and NL groups. Their ROC curves were further used to evaluate their diagnostic values for AAV patients complicated with a lung infection. The results showed that all indicators except ESR showed good sensitivity and specificity. Among them, PCT has the highest AUC value, with a sensitivity of 85.3% and specificity of 84%. CRP had the second-highest diagnostic value, while ESR had the worst diagnostic value for infection with a specificity of only 54%. To further improve the diagnostic efficiency, we calculated the composite indicator of all possible biomarkers, and the results showed that the composite indicator of PCT-CRP-NLCR had the highest diagnostic value.

The Birmingham vasculitis activity score (BVAS) was initially proposed by Luqmani et al. [31] and later revised [13, 32]. In this system, BVAS > 15 points indicates disease activity, and the higher the BVAS, the higher the disease activity. Studies have shown that BVAS at onset is an independent risk factor for pulmonary lesions of AVV patients [33]. However, other studies also show that BVAS is not highly sensitive to AAV activity and hard to distinguish between organ damages caused by vasculitis activity or inactivity in clinical practice. In this study, the average BVAS was 19.06 for patients in the LI group, significantly higher than that of less than 15 for patients in the NL and NI groups, suggesting that AAV patients are more prone to lung infection.

## Supplementary Information

**Additional file 1.** More clinical features of the subjects.

## Data Availability

The data that support the findings of this study are available from the corresponding author upon reasonable request.
