# Supplementary material for: Clinical features and markers to identify pulmonary lesions caused by infection or vasculitis in AAV patients
Source: BMC Pulm Med. 2023 Jan 18;23:27. doi: 10.1186/s12890-023-02317-7 (PMC9850570; doi:10.1186/s12890-023-02317-7)
Supplement: Supplementary file 1 — Additional file 1. More clinical features of the subjects. [file 12890_2023_2317_MOESM1_ESM.docx]

Supplementary Table 1. Frequency of clinical manifestations in 140 AAV patients captured by the Birmingham Vasculitis Activity Score (BVAS).

| **BVAS items (by organ system)** | **Number of patients with symptoms** |
| --- | --- |
| **General** (Maximum allowable score:3. Mean score: 0.28) |  |
| Malaise | 0 |
| Myalgia | 0 |
| Arthralgia/arthritis | 2 |
| Headache | 0 |
| Fever (<38.5 degrees Celsius) | 12 |
| Fever (>38.5 degrees Celsius) | 24 |
| Weight loss (≥2 kilograms) | 1 |
| **Cutaneous** (Maximum allowable score:6. Mean score: 0.09) |  |
| Infarct |  |
| Purpura | 3 |
| Gangrene | 1 |
| Ulcer | 0 |
| Other skin vasculitis | 0 |
| **Mucous membranes/eyes** (Maximum allowable score:6. Mean score: 0.07) |  |
| Mouth ulcers | 0 |
| Genital ulcers | 0 |
| Adnexal inflammation | 0 |
| Significant proptosis | 0 |
| Red eye (Epi)scleritis | 1 |
| Red eye conjunctivitis/ | 1 |
| Blurred vision | 0 |
| Sudden vision loss | 0 |
| Uveitis | 0 |
| Retinal vasculitis/retinal vessel | 1 |
| Thrombosis/retinal exudates/ retinal haemorrhages | 0 |
| **Ear, Nose, and Throat (**Maximum allowable score:6; Mean score: 0.41) |  |
| Bloody nasal discharge/nasal crusts/ulcers and/or granulomata | 2 |
| Paranasal sinus involvement | 8 |
| Subglottic stenosis | 1 |
| Conductive hearing loss | 6 |
| Sensorineural hearing loss | 0 |
| **Chest (**Maximum allowable score:6. Mean score: 3.29) |  |
| Wheeze | 45 |
| Nodules or cavities | 50 |
| Pleural effusion/pleurisy | 42 |
| Infiltrate | 52 |
| Endobronchial involvement | 17 |
| Massive haemoptysis/Alveolar haemorrhage | 23 |
| Respiratory failure | 4 |
| **Cardiovascular (**Maximum allowable score:6. Mean score: 0.56) |  |
| Loss of pulses | 0 |
| Valvular heart disease | 0 |
| Pericarditis | 0 |
| Ischaemic cardiac pain | 3 |
| Cardiomyopathy | 3 |
| Congestive heart failure | 10 |
| **Abdominal (**Maximum allowable score:9; Mean score: 0.51) |  |
| Peritonitis | 9 |
| Bloody diarrhoea | 6 |
| Ischaemic abdominal pain | 1 |
| **Renal (**Maximum allowable score:12; Mean score: 10.63) |  |
| Hypertension | 92 |
| Proteinuria >1+ | 118 |
| Haematuria ≥10 rbc/hpf | 110 |
| Creatinine 125-249 µmol/l | 26 |
| Creatinine 250-499 µmol/l | 38 |
| Creatinine ≥ 500 µmol/l | 33 |
| Rise in creatinine > 30% or creatinine clearance fall > 25% | 13 |
| **Nervous system (**Maximum allowable score:9. Mean score: 0.15) |  |
| Headache | 0 |
| Meningitis | 0 |
| Organic confusion | 4 |
| Seizures (not hypertensive) | 1 |
| Stroke | 0 |
| Cord lesion | 0 |
| Cranial nerve palsy | 0 |
| Sensory peripheral neuropathy | 0 |
| Motor mononeuritis multiplex | 0 |
| **Other** | 0 |

Supplementary Table 2. Category of pathogens in patients of LI group.

| Pathogens | Number of patients, n (%) |
| --- | --- |
| Gram-positive bacteria |  |
| *Enterococcus faecium* | 1 (2.38) |
| *Streptococcus* | 14 (33.33) |
| *Corynebacterium* | 1 (2.38) |
| *Staphylococcus aureus* | 3 (7.14) |
| *Streptococcus granulosus* | 2 (4.76) |
| *Staphylococcus glycolyticus* | 1 (2.38) |
| *Gemella haemolysans* | 1 (2.38) |
| *Lactobacillus Rossi* | 1 (2.38) |
| Gram-negative bacteria |  |
| *Escherichia coli* | 2 (4.76) |
| *Haemophilus parainfluenzae* | 1 (2.38) |
| *Acinetobacter baumannii* | 1 (2.38) |
| *Neisseria* | 3 (7.14) |
| *Acinetobacter lwoffi* | 2 (4.76) |
| *Citrobacter* | 1 (2.38) |
| Fungus |  |
| *Candida albicans* | 4 (9.52) |
| *Candida parapsilosis* | 1 (2.38) |
| *Aspergillus* | 3 (7.14) |
| Total |  |
|  | 42 (100) |

Supplementary Table 3. Duration of symptoms

| Symptoms | Duration of symptoms (months) | | |
| --- | --- | --- | --- |
|  | NI  n=50 | LI  n=68 | *p* value |
| Weakness | 0.73±1.01 | 1.02±1.98 | 0.415 |
| Chest tightness | 0.51±0.97 | 0.78±1.37 | 0.562 |
| Cough and expectoration | 0.58±0.96 | 0.87±1.37 | 0.505 |
| Hemoptysis | 0.15±0.45 | 0.38±1.10 | 0.097 |
| Fever | 0.03±0.12 | 0.46±0.78 | 0.001* |
| Edema | 0.35±0.62 | 0.36±0.68 | 0.821 |

Values are expressed as number (percentage). **p*<0.05.
